# Allometric approach to the two male morphs in the Japanese firefly *Luciola parvula*

**DOI:** 10.3389/finsc.2023.1230363

**Published:** 2023-07-24

**Authors:** Yutaka Iguchi

**Affiliations:** Laboratory of Biology, Okaya, Nagano, Japan

**Keywords:** body size, variation, discontinuity, phenotypic plasticity, dimorphism

## Introduction

*Luciola parvula* (Coleoptera, Lampyridae) is a terrestrial firefly species widely distributed in Japan and shows large geographical variation in male adult body size especially in mountainous areas. Previous studies reported that there were two male morphs of adults of this species: large and small morphs. However, the procedure used to identify the two male morphs has remained ambiguous and confusing. Therefore, the present article begins with a brief review of the two male morphs in this species and then proposes an allometric approach to support the existence of the two male morphs.

As far as the present author knows, Ohba (1) was the first to describe the criteria for the classification of the two male morphs in this species. He collected male adults at Mt. Hakone, central Japan and then identified the large morph above 700 m altitude and the small morph below this altitude. He also reported that the approximate body length was 10 mm for the large morph and 6 mm for the small morph.

Ohba (2) further examined male adults of this species throughout Japan and then found the large morph with body length 7 to 9 mm and pronotum width larger than 2.1 mm and the small morph with body length 5 to 7 mm and pronotum width smaller than 2.1 mm.

Mitsuishi (3–6) collected male adults of this species at 41 sites in Nagano Prefecture, central Japan and classified them into the large morph (body length larger than 8 mm) and the small morph (body length approximately 7 mm). As a result, he found that this species in this prefecture tends to be segregated into a northeastern and a southwestern group corresponding to the large and the small morph, respectively, but that both morphs exist above 1000 m altitude. However, his definition of the size of the two morphs was different from Ohba’s definition (2) (large male morph with body length 7 to 9 mm and small morph with body length 5 to 7 mm). In other words, Mitsuishi’s specimens (3–6) rather belonged to the large male morph according to Ohba’s definition (2).

Mitsuishi (3–6) explained neither why he used his own definition of the two morphs, nor how he determined the boundary of the two morphs. He showed mean values of his measurements, but did not perform further statistical analysis. Therefore, the present article aims to reanalyze Mitsuishi’s data (3–6) and examine the existence of the two male morphs using cluster and allometric analysis as mentioned below in detail.

Ohba (7) reviewed his studies on the two male morphs of this species and summarized his criteria for the classification of them: the large morph with body length 7 to 8 mm below 1600 m altitude and the small morph with body length 5 to 6 mm below 800 m altitude.

Kusaoke et al. (8) investigated the distribution of this species in Toyama Prefecture, adjacent to Nagano Prefecture mentioned above and found the small male morph (body length approximately 6 mm) at three sites at altitudes of 400 to 700 m and the large male morph (body length approximately 8 mm) at an altitudes of 1100 m. As they mentioned, these findings almost fell into Ohba’s criteria (7). Therefore, they considered that Mitsuishi (3–6) had found only the large male morph above 1000 m altitude in Nagano Prefecture.

However, Kusaoke et al. (8) also found at Mt. Daisen, Tottori Prefecture, that the large male morph (mean body length 8.12 mm) exited at 1709 m altitude and small male morph (mean body length 7.14 mm) at 800 m altitude. These mean values of body length were not consistent with Ohba’s criteria (7).

In these studies, males of this species were more or less arbitrarily classified into the large and small morphs. No statistical analysis was performed to examine whether or not body size varies continuously.

Molecular phylogenetic studies suggested that the small male morph originated from an ancestor similar to the large male morph (9, 10). However, no genetic difference was found between the two male morphs at Mt. Daisen, Tottori Prefecture (8).

Ohba (7) also showed that the flash interval (0.5 to 0.6 s) of the small male morph is smaller than that (0.7 to 0.8 s) of the large male morph. However, he did not take into consideration the effect of temperature on flash intervals. In fact, such temperature effects were found and statistically analyzed in *Nipponoluciola cruciate* (formerly *Luciola cruciate*) (11–13). Therefore, it remains unclear whether the two male morphs of this species show different flash intervals under controlled temperature.

Regarding morphological measurements of *L*. *parvula*, the previous studies focused on simple descriptive statistics such as mean values of body length. However, multivariate analyses are considered necessary for detecting size dimorphism in this species. Therefore, the existence of inconsistent criteria for the two male morphs of this species leads the present author to reexamine published data on measurements of this species.

Fortunately, Mitsuishi (3–6) showed his measurement data on male body length, body width and pronotum width at 41 sites in Nagano Prefecture. Using these data, the present article attempts to detect differences in scaling relationships between the two male morphs by allometric analysis. The results will help to provide direction for future studies on the male size dimorphism of this species.

## Materials, methods, and analysis

The data analyzed here were obtained from Mitsuishi’s data (3–6) published in Zenkoku Hotaru Kenkyukai-shi (Proceedings of the Japan Association for Fireflies Research). These articles are freely available on the website of the Japan Association for Fireflies Research (http://zenhoken-std.sakura.ne.jp/). Mitsuishi (3–6) recorded his measurement data as the mean values of male body length (length from anterior margin of pronotum to posterior margin of elytra), body width (width across both elytra covering mesonotum) and pronotum width at 41 sites in Nagano Prefecture.

**Figure 1:**
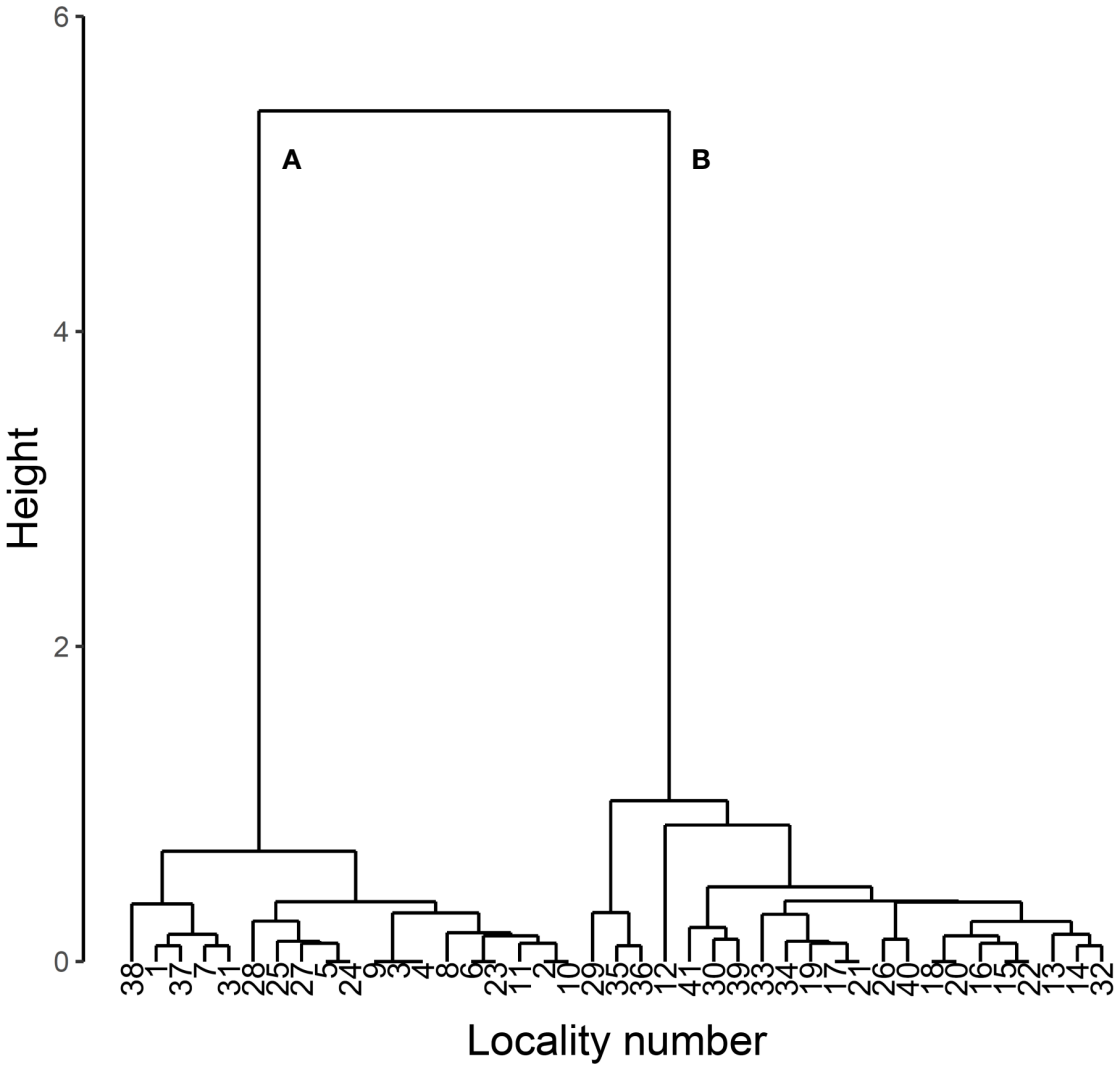
Dendrogram of cluster analysis of body length, body width and prothorax width in *L*. *parvula*. The analysis was performed using Ward’s method with Euclidean distance. The data were obtained from Mitsuishi (3–6). Two major clusters are labeled as **(A)** and **(B)**.

The present study first classified the 41 specimen data set into two groups by hierarchical cluster analysis with the function hclust of R version 4.2.2 (14) using Ward’s method with Euclidean distance. As a result, two major groups were identified (**Figure 1**).

Next, allometric equations were applied to the two groups using standardized major axis regression with the smatr package in R version 4.2.2 (14). The data were log_10_ transformed and thereby the log-log relationship between pronotum width (*x*) and body length (*y*), the log_10_-transformed allometric equation was expressed as:


$$log_{10}y=log_{10}a+b\;log_{10}x$$

where *a* and *b* are constants. The constant *b* is the slope of the line, also known as the allometric coefficient.

**Figure 2:**
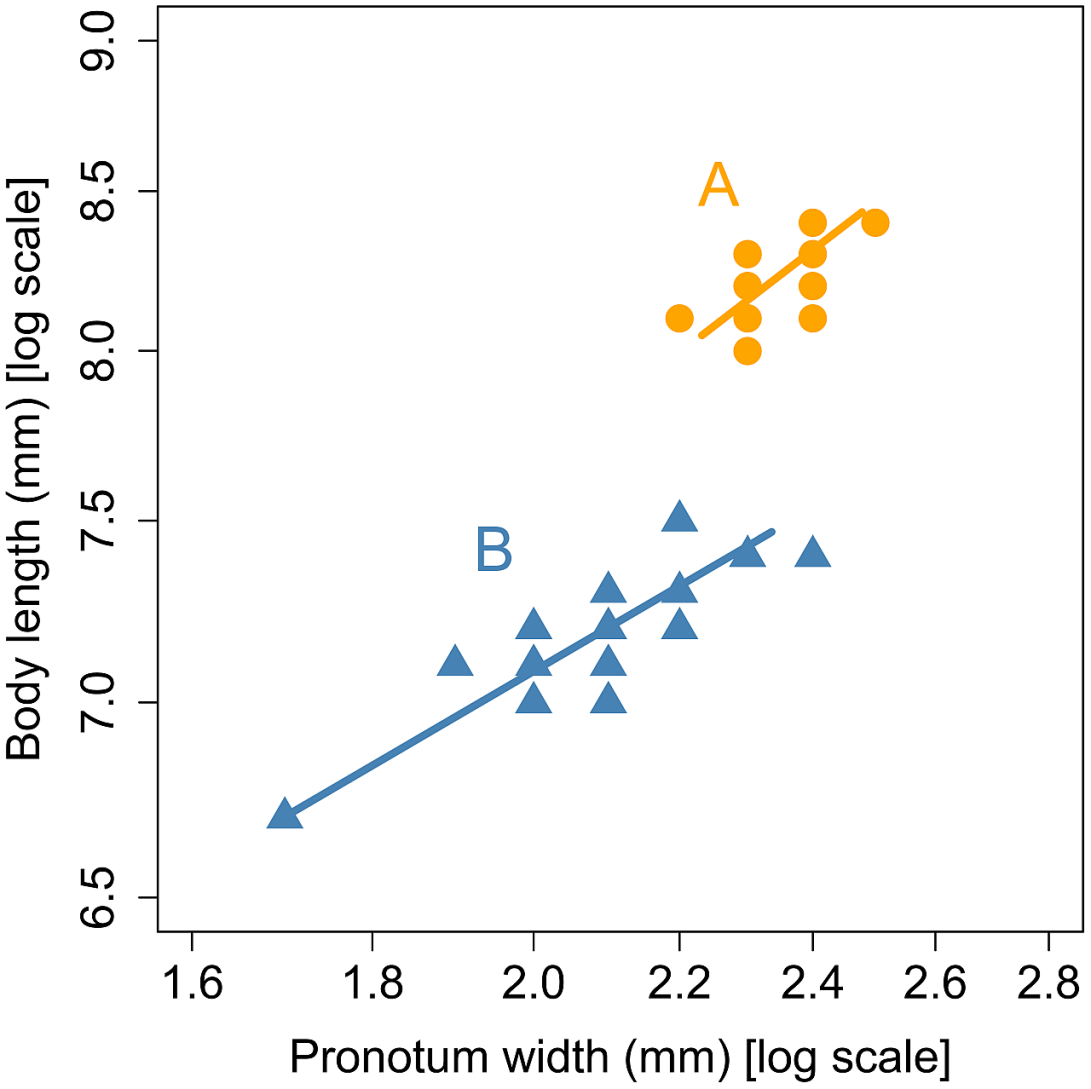
Allometric relationship between pronotum width and body length in the groups **(A)** and **(B)** in male *L*. *parvula* fireflies. The groups were identified by the cluster analysis of Mitsuishi’s published data (3–6). The standardized major axis regression lines were separately fitted to each group.

This allometric equation was fitted to each of the two groups identified by the cluster analysis. As shown in **Figure 2**, the two allometric lines representing the two groups did not differ significantly in slope (*χ*2 = 0.077, df = 1, *p* = 0.78), but did differ significantly in elevation (*χ*2 = 229.6, df = 1, *p*< 0.001). The two allometric lines were found to be parallel and share a common slope of 0.34.

## Discussion

Kusaoke et al. (8) followed Ohba’s criteria (small male morph, body length 5 to 6 mm, below 800 m altitude) (7) and suggested the possibility that almost all the specimens collected by Mitsuishi (3–6) may belong to the large male morph. However, the present article confirmed the existence of the two male morphs showing discontinuous allometric scaling relationship.

Kusaoke et al. (8) found no genetic difference between the two male morphs at Mt. Daisen, Tottori Prefecture. Moreover, Suzuki (9) and Hiyori et al. (10) reported no genetic difference between them in Nagano Prefecture where Mitsuishi (3–6) collected the specimens. Therefore, in *L*. *parvula*, the difference in allometry between the two morphs with genetic similarity may be connected with the morphological plasticity of this species.

Such morphological plasticity is well known in many animal species [reviewed in (15)]. Kusaoke et al. (8) suggested that the large male morph of *L*. *parvula* shows better adaptation to high altitudes than the small male morph. However, Mitsuishi’s data (3–6) analyzed here showed the existence of both morphs at high altitudes.

The allometric analysis of Mitsuishi’s data (3–6) revealed that the two male morphs of *L*. *parvula* show different allometric patterns. Such allometric variation was observed in experimental studies on coleopteran insects such as horned beetles (16–18). These studies showed that allometric relationships in adults vary depending on larval nutrition. However, such studies have not yet been performed on *L*. *parvula*. Therefore, it remains unclear about the relationship between adult body shape and larval nutrition in males of this species.

Male dimorphism is scarcely studied in fireflies except for *Abscondita cerata* (previously named *Luciola cerata*) in Taiwan (19). The two male morphs of this species show different mating strategies (19). However, it remains unclear whether male *L*. *parvula* show different mating strategies between the two morphs.

There are two major limitations of this article. First, the analyzed data sources were restricted to specimens collected in Nagano Prefecture. This species is widely distributed in Japan. Therefore, specimens collected in other areas will be required to be statistically analyzed in future studies.

The second limitation is that the analyzed data were restricted to males. Ohba (20) collected females of this species at Mt. Hakone and the moat of Nagoya Castle and then measured their pronotum width and length. As a result, he reported difference in mean size of females between the two sites and thereby identified the existence of female size dimorphism. However, he performed no statistical test of the difference. Female morphological data are very scarce in this species. Therefore, it remains unclear about the existence of female size dimorphism.

In conclusion, the present article strongly recommends that allometric analysis should be used to test the presence of the two morphs in both males and females in this species in future studies.

## Author contributions

The author confirms sole responsibility for the following: study conception and design, data collection, analysis and interpretation of results, and manuscript preparation.
